# Establishment of a model for predicting the outcome of induced labor in full-term pregnancy based on machine learning algorithm

**DOI:** 10.1038/s41598-022-21954-2

**Published:** 2022-11-09

**Authors:** Tingting Hu, Sisi Du, Xiaoyan Li, Fang Yang, Shanshan Zhang, Jingjing Yi, Birong Xiao, Tingting Li, Lin He

**Affiliations:** 1 People’s Hospital of Deyang City, Deyang, 618000 Sichuan China; 2 School of Nursing, Wenzhou Medical University, Wenzhou, 325035 Zhejiang China; 3 The First Affiliated Hospital of Wenzhou Medical University, Wenzhou, 325000 Zhejiang China

**Keywords:** Computational biology and bioinformatics, Medical research

## Abstract

To evaluate and establish a prediction model of the outcome of induced labor based on machine learning algorithm. This was a cross-sectional design. The subjects were divided into primipara and multipara, and the risk factors for the outcomes of induced labor were assessed by multifactor logistic regression analysis. The outcome model of labor induced with oxytocin (OT) was constructed based on the four machine learning algorithms, including AdaBoost, logistic regression, naive Bayes classifier, and support vector machine. Factors, such as accuracy, recall, precision, F1 value, and receiver operating characteristic curve, were used to evaluate the prediction performance of the model, and the clinical application of the model was verified. A total of 907 participants were included in this study. Logistic regression algorithm obtained better results in both primipara and multipara groups compared to the other three models. The accuracy of the model for the prediction of “successful induction of labor” was 94.24% and 96.55%, and that of “failed induction of labor” was 65.00% and 66.67% in the primipara and the multipara groups, respectively. This study established a prediction model of OT-induced labor based on the Logistic regression algorithm, with rapid response, high accuracy, and strong extrapolation, which was critical for obstetric clinical nursing.

## Introduction

Induction of labor in full-term pregnancy refers to using drugs and other means to induce labor for delivery. It is one of the most common methods to deal with high-risk pregnancy in obstetrics when natural childbirth cannot be initiated spontaneously^1^. Oxytocin (OT) has extensive clinical application, is widely used, can be controlled easily, and is the safest induced labor drug that can be utilized in all pregnant women who need labor induction. Typically, full-term induced labor is one of the major challenges in obstetrics, and the success of labor induction is the main factor affecting fetal survival^2^.

Presently, Bishop score is commonly used for the evaluation of the outcomes of induced labor, including cervical conditions and evaluating cervix orientation, cervix texture, uterine orifice dilation, and fetal prolapse^3^. However, this method has limitations in OT-induced labor. It can only indicate the situation of the vagina cervix but cannot reflect the overall labor progress of pregnant women. In addition, differences were detected due to individual subjective factors and clinical experience; Bishop score was used to evaluate the success of induced labor, which presented the disadvantages of strong subjectivity and error. Some studies found that Bishop score is a successful standard of induced labor but has many disadvantages^4^. Therefore, in the present study, we aimed to find effective means and various clinical indicators and improve the accuracy of prediction in the induction of labor.

Literature review found the limitations of previous studies as follows: ① First, there was a lack of accurate and systematic early prediction of the outcome of OT-induced labor. Several studies showed that the traditional Bishop score was not suitable for the current situation of obstetrical diagnosis and treatment. ② Second, previous studies had some defects, such as sample size and lack of inclusion of prediction factors, which affected the accuracy of the model. ③ Some studies proposed using ultrasound parameters, cervical elastography, and fetal fibronectin to predict the probability of successful labor induction, which improved the accuracy of the model. However, these variables were not available to all medical institutions; hence, the clinical application of the prediction model was limited. ④ In addition, most of the prediction models reported in the literature were based on the traditional logistic regression method. However, many indexes affecting the outcome of induced labor presented a complex intercorrelation. To predict the success of induced labor, the integrity, complexity, and dynamics of the labor process and the nonlinear synergy among various risk factors need to be considered. It is difficult for the traditional statistical early warning model to meet these requirements. Machine learning algorithms have many advantages in dealing with multivariate nonlinear data^5^, while no studies have applied machine learning to the outcome of induction of labor with OT.

With the development of artificial intelligence and medical information technology, a large number of machine learning algorithms have been applied to assess the disease risk of prediction models. For example, some studies applied machine learning technology to the risk of delirium^6^, nursing medication errors^7,8^, readmission to the intensive care unit (ICU)^9^, pressure sores, fall^10–12^, and even depression suicides^13,14^, and achieved satisfactory results, thereby proving that the model based on machine learning was effective.

This study aimed to explore the factors affecting the outcome of OT-induced labor. The population was divided into primipara and multipara groups, and the prospective prediction of the outcome of induced labor was based on the machine learning algorithm to provide a basis for consultation, classification, risk stratification, and delivery plan before OT induction.

## Methods

### Participants

This was a retrospective cohort study design. The pregnant participants used OT-induced labor during delivery in the Obstetrics Department of the First Affiliated Hospital of Wenzhou Medical University China from January 2019. The participants were divided into two groups according to the number of births: primipara and multipara.

The inclusion criteria of this study were as follows: Gestational age was ≥ 37 weeks, less < 41 weeks; No serious pregnancy complications; Induction of labor with OT; Patients and their families had informed consent to the scheme of OT-induced labor.

The exclusion criteria were as follows: Contraindications to vaginal delivery (such as cephalopelvic disproportion, severe contracted pelvis, abnormal fetal position, fetal distress, and placenta previa); Contraindications for the use of OT (such as abnormal fetal position, obvious cephalopelvic disproportion, antepartum hemorrhage, and pregnancy complicated with severe cardiopulmonary insufficiency); incomplete clinical data.

### Predictors and data preprocessing

The literature review and the knowledge of a panel of experts identified 18 factors that might affect the outcome of OT-induced labor, including age, height, weight, body mass index, gestational age, number of cesarean sections, number of abortions, Bishop score, fetal weight, amniotic fluid index, amniotic fluid contamination, B-mode ultrasound data (fetal head circumference, fetal abdominal circumference, fetal biparietal diameter, and fetal bone length), maternal uterine height, maternal abdominal circumference, fetal membrane status, and labor analgesia. These data were retrieved from the electronic medical record system.

Among them, age, height, and the other 13 variables were numerical variables, while labor analgesia, fetal membrane state, and amniotic fluid contamination were dichotomous variables. The assigned values were input, as shown in Table 1.

**Table 1:** Maternal characteristics among induction of labor.

Characteristics	Primipara (n = 495)			Multipara (n = 312)		
	Success (n = 419)	Failure (n = 76)	*P*-value	Success (n = 300)	Failure (n = 12)	*P*-value
Mean age, years	27.04 ± 3.24	27.97 ± 2.98	0.072	31.11 ± 4.52	36.33 ± 4.79	0.023
Mean height, m	1.61 ± 0.05	1.59 ± 0.05	0.067	1.60 ± 0.05	1.55 ± 0.06	0.064
Mean weight, kg	67.82 ± 9.11	69.63 ± 9.00	0.088	69.33 ± 8.95	65.96 ± 7.92	0.148
Body mass index, kg/m^2^	25.53 ± 3.43	26.21 ± 3.39	0.838	26.10 ± 3.37	24.83 ± 2.98	0.147
Gestational age, weeks	39.62 ± 0.93	39.96 ± 0.90	0.341	39.43 ± 1.12	39.46 ± 1.55	0.202
Number of abortions	0.33 ± 0.60	0.46 ± 0.89	0.99	0.94 ± 1.13	1.33 ± 1.07	0.237
Fetal weight	3385.08 ± 1585.27	3564.74 ± 409.54	0.895	3420.4 ± 429.65	3536.67 ± 570.60	0.697
Amniotic fluid index	92.10 ± 32.65	97.54 ± 33.86	0.724	98.43 ± 37.57	104.5 ± 29.77	0.65
Head circumference	333.34 ± 10.89	336.53 ± 10.29	0.284	333.36 ± 13.88	320.42 ± 64.79	0.046
Abdominal circumference	341.77 ± 17.31	351.13 ± 16.11	0.028	344.65 ± 20.03	350.33 ± 17.97	0.061
Bone length	72.72 ± 3.34	72.86 ± 6.29	0.007	72.55 ± 4.16	72.33 ± 4.23	0.73
Maternal uterine height	34.57 ± 1.96	35.53 ± 2.31	0.295	34.97 ± 2.45	35.42 ± 2.43	0.5
Maternal abdominal circumference	97.63 ± 19.75	98.57 ± 6.19	0.878	99.36 ± 17.75	99.5 ± 6.01	0.087
Fetal biparietal diameter	93.10 ± 3.52	93.96 ± 3.13	0.654	92.53 ± 4	93.42 ± 2.91	0.273
Bishop score	3.38 ± 1.92	1.40 ± 1.84	< 0.001	2.69 ± 1.69	0.5 ± 0.67	0.018
Amniotic fluid contamination		< 0.001		0.926		
Contaminated	6 (46.2%)	7 (53.8%)		8 (88.9%)	1 (11.1%)	
Uncontaminated	413 (85.7%)	69 (14.3%)		292 (96.4%)	11 (3.6%)	
Fetal membrane state			0.351			0.218
broken	161 (89.9%)	18 (10.1%)		100 (98.0%)	2 (2.0%)	
Unbroken	258 (81.6%)	58 (18.4%)		200 (95.2%)	10 (4.8%)	
Labor analgesia			0.26			0.342
Yes	354 (85.5%)	60 (14.5%)		137 (95.1%)	7 (4.9%)	
No	65 (80.2%)	16 (19.8%)		163 (97.0%)	5 (3.0%)	

### Induced labor scheme

All participants were treated with low-dose OT induction or combined with cook balloon, as described below: A doctor assessed the condition of the pregnant woman’s pelvis and cervix. If Bishop score was < 6, cervical maturation was induced by cook balloon, and uterine contractions were observed. If uterine contractions were irregular, OT (2.5 U) was added into 0.5% normal saline (NS) (500 mL) by an intravenous drip (flow rate 5 drops/min, maximum drop rate 40 drops/min). If Bishop score was > 6, the uterine contractions, OT, and drop rate conditions were the same as above.

### Outcomes

The participants were divided into primipara and multipara groups. The dependent variable was the outcome of induction of labor and was dichotomized into binary outcomes as 0 and 1. Thus, the successful induction of labor was recorded as 1, and failed induction of labor was recorded as 0. The successful of induction of labor is defined as delivery within 3 days post-administration of OT, while failed induction of labor was defined as no indication of labor after 3 days.

### Screening of modeling variables

In this study, the non-conditional logistic regression method was used to select statistically significant independent variables by univariate and multivariate analysis; OT induction was the dependent variable, and 18 suspected factors of OT induction were independent variables. For logistic regression and naive Bayes models in this study, single factor analysis was performed first, and the results are shown in Table 1. The variables with statistical significance (*P* < 0.10) were analyzed by non-conditional multivariate analysis, and those with statistical significance (*P* < 0.10) in the non-conditional multivariate analysis were included as modeling variables (Table 2).

**Table 2:** Results of multivariate logistic stepwise regression analysis.

Factors	Regression coefficient	Standard error	*P*	OR
**Primiparas**				
Age	− 0.082	0.045	0.066	0.921
Height	7.306	3.752	0.052	1489.05
Weight	− 0.034	0.02	0.088	0.967
Amniotic fluid contamination	3.547	0.924	< 0.001	34.718
Abdominal circumference	− 0.046	0.011	< 0.001	0.955
Bone length	0.076	0.036	0.033	1.079
Bishop score	0.909	0.133	< 0.001	2.482
**Multiparas**				
Age	− 0.324	0.097	0.001	0.723
Height	29.396	11.1	0.008	5.84e + 12
Head circumference	0.03	0.015	0.049	1.03
Abdominal circumference	− 0.06	0.033	0.072	0.942
Bishop score	2.975	0.86	0.001	19.589

### Establishing OT-induced labor outcome prediction model based on machine learning algorithms

Four machine learning algorithms, including logistic regression, naive Bayes, support vector machine (SVM), and AdaBoost algorithm, were used to establish early warning models of OT-induced labor outcomes in primipara and multipara. The grid search method (GridsearchCV) was used to adjust the optimal parameters. The core parameters of the AdaBoost model were set as follows: the number of iterations was set to 200, the selected learning rate was 0.1, the number of learners was set to 30, the maximum number of classes was set to 20, and other parameters were set at default values. The core parameters of the logistic regression model were as follows: the function fitglm for training, the number of classifiers was 10, the maximum number of leaf nodes was none, the minimum impurity of node division was 10^−7^, and the sampling method was bootstrap. Both naive Bayes model and SVM used Gaussian radial kernel function (RBF) to train and predict the data.

### Evaluation of the prediction model

The area under a curve (AUC) of a receiver operating characteristic (ROC) curve, sensitivity, specificity, and F1 score were used to evaluate the performance of the model.

### Clinical prospective verification of the prediction model

The prediction model with the best performance in the two groups was selected for external validation, and the data from pregnant women who used OT for labor induction in the same hospital from January to March 2020 were collected. The difference between the actual clinical outcome of labor induction and the decision result of the prediction model was compared and expressed by accuracy rate (%).

## Statistical method

SPSS22.0 was used for data analysis. The 18 influencing factors and the outcome of OT-induced labor were analyzed by logistic single factor analysis. According to the single factor analysis results, a stepwise regression method was used to screen the variables; *P* < 0.1 means the difference was statistically significant. Establish and verify different types of machine learning algorithms based on MATLAB 2019B.

## Ethics approval

The experimental protocols were approved by the Ethics Committee of Wenzhou Medical University (No. 2019089). We confirmed that all methods were carried out in accordance with the relevant guidelines, and written informed consent was obtained from all participants included in the study.

## Results

### Baseline characteristics

**Figure 1:**
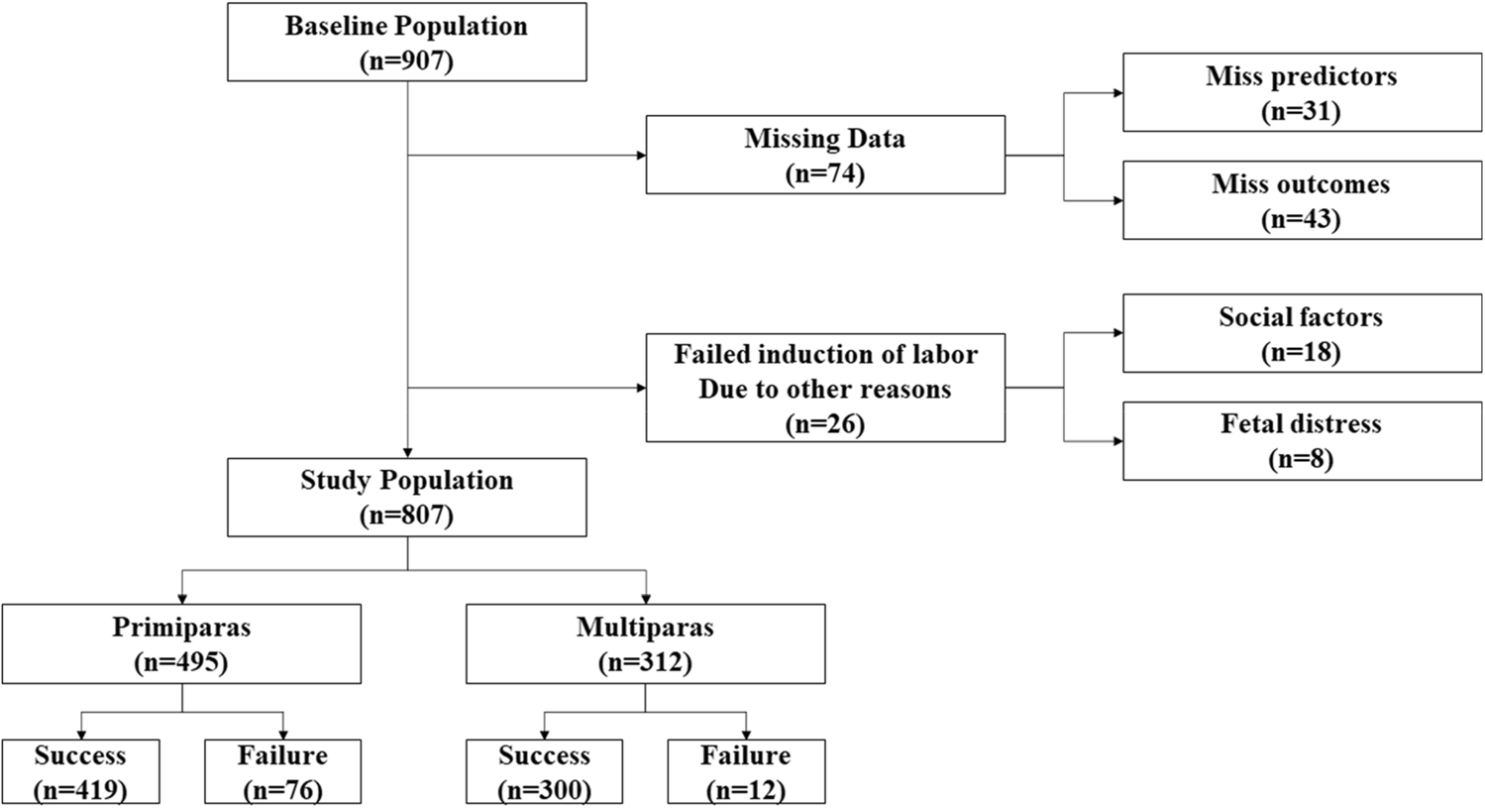
A flow chart for study population selection.

A total of 907 participants were included in this study. After excluding 74 participants with missing data (31 for predictors, 43 for outcomes), excluding 26 participants with failed induction of labor due to other reasons [13 participants (social factors) and 8 participants (fetal distress)], 495 primiparas and 312 multiparas comprised the cohort of this study (Fig. 1).

Table 1 shows the stratification of 495 primiparas and 312 multiparas by induced labor outcome.

### Identification of feature importance

For primiparas, the results of the univariate analysis showed statistically significant differences in age, height, weight, amniotic fluid contamination, abdominal circumference, bone length, and Bishop score (*P* < 0.10). For multiparas, the results of univariate analysis showed statistically significant differences in age, height, head circumference, abdominal circumference, and Bishop score (*P* < 0.10). The variable assignment and results of univariate regression analysis are shown in Table 1.

Then, the statistically significant factors were considered independent variables for the non-conditional multivariate analysis. The results showed that statistically significant variables in univariate analysis were independent influencing factors of OT-induced labor outcome. The results of multivariate regression analysis are shown in Table 2.

### Comparison of the results of OT-induced labor outcome prediction models based on four machine learning algorithms

Logistic regression algorithm obtained better results in both the primipara and multipara groups compared to the other three models. In the primipara group, the results showed that the logical regression model with an accuracy of 0.903, the recall rate was 0.986, the precision rate was 0.908, and the F1 value was 0.943. In the multipara group, the model with an accuracy of 0.971, the recall rate was 0.993, the precision rate was 0.977, and the F1 value was 0.982 (Table 3).

**Table 3:** Accuracy, precision, recall rate, and F1 value of the four models for predicting the outcome of induced labor.

Model	Primiparas				Multiparas			
	Accuracy	Recall	Precision	F1	Accuracy	Recall	Precision	F1
Logistic regression	0.903	0.986	0.908	0.943	0.971	0.993	0.977	0.982
Naive Bayes	0.84	0.976	0.856	0.903	0.955	0.987	0.967	0.971
SVM	0.853	1	0.852	0.92	0.962	1	0.962	0.98
Adaboost	0.901	0.969	0.919	0.934	0.962	1	0.962	0.98

**Figure 2:**
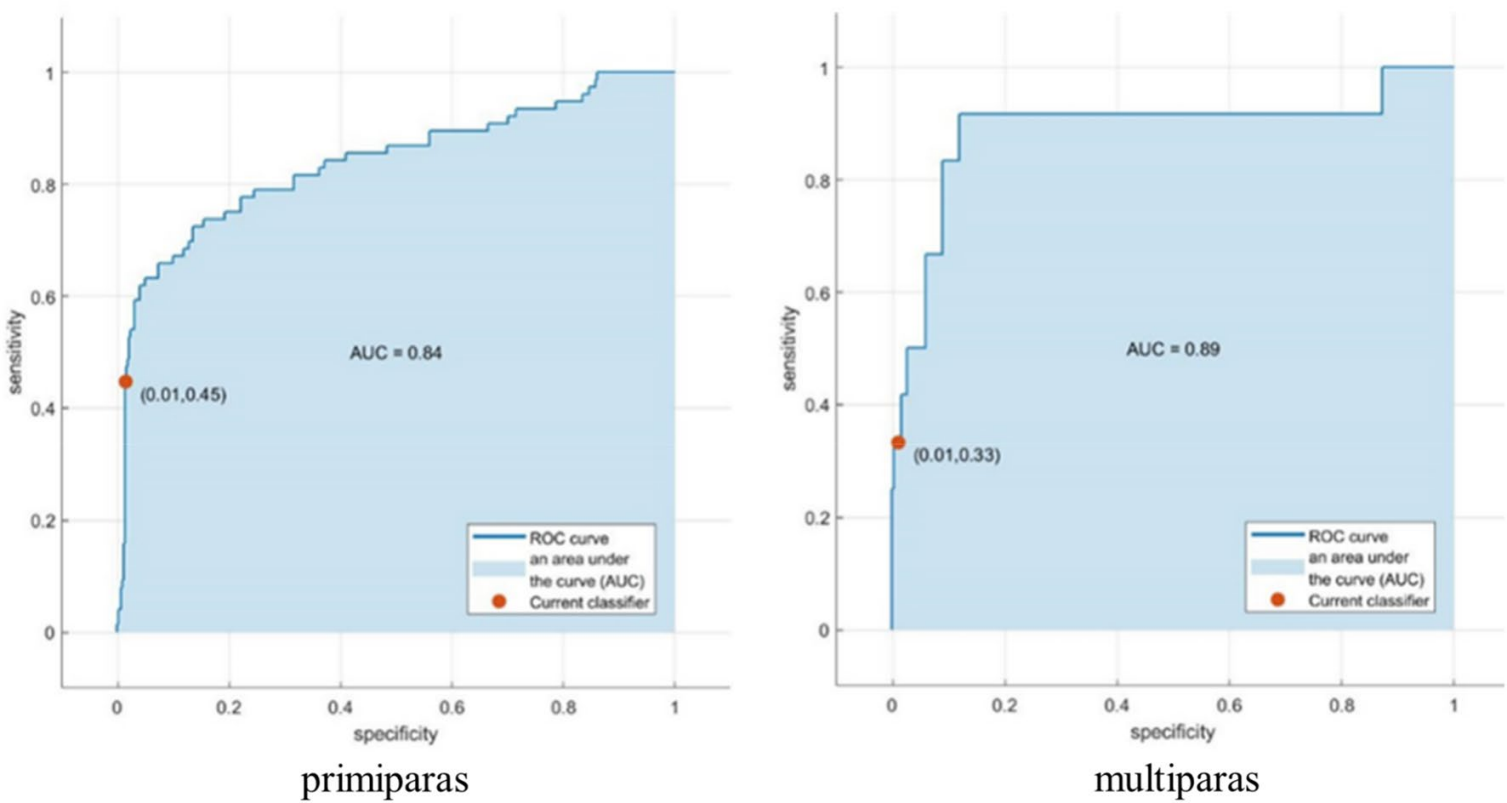
ROC curves of logistic regression model of primiparas and multiparas.

The AUC of the logistic regression model of primiparas and multiparas was 0.84 and 0.89, respectively (Fig. 2).

### Model verification results

The optimal logistic regression algorithm was selected for external clinical validation. The cohort consisted of 159 OT-induced primiparas from January to March in 2020. Among the 159 cases, induction of labor was successful in 139 cases but failed in 20 cases. The accuracy of the model for the prediction of “successful induction of labor” was the highest, reaching 94.24%, and that of “failed induction of labor” was 65.00%. In the multipara group, the logistic regression prediction model was clinically validated using 96 OT-induced multiparas from January to March in 2020. Among the 96 cases, induction of labor was successful in 87 cases and failed in 9 cases. The accuracy of the model for the prediction of “successful induction of labor” was the highest, reaching 96.55%, followed by the prediction of “failed induction of labor” (66.67%). The results of the confusion matrix are shown in Table 4.

**Table 4:** External verification results of prediction model.

Actual outcome of induction of labor	Primipara prediction model			Multipara prediction model		
	Success	Failure	Accuracy rate (%)	Success	Failure	Accuracy rate
Success	131	8	94.24	84	3	96.55%
Failure	7	13	65.00	3	6	66.67%

### Ranking of model variables

**Figure 3:**
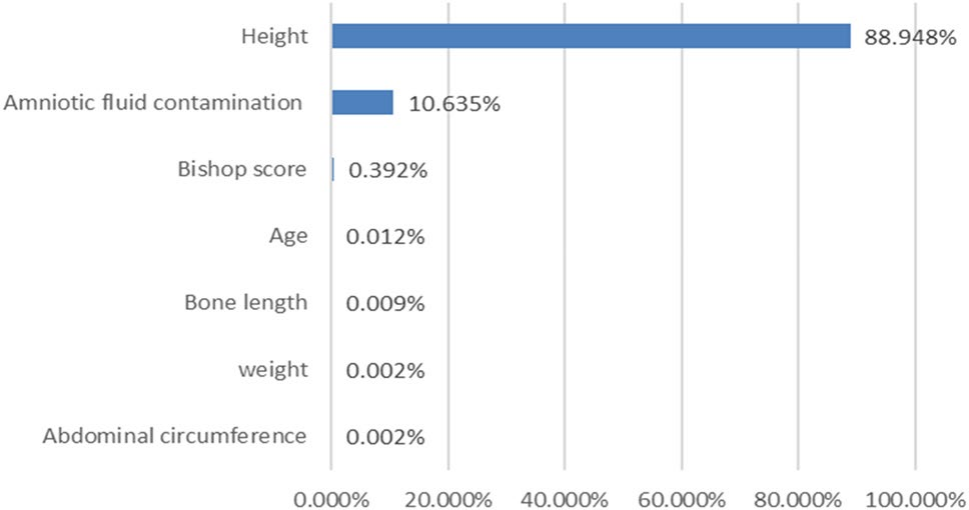
Ranking of the variables of logistic regression model of primiparas.

The distribution of the variables incorporated in the logistic regression prediction model of primiparas is shown in Fig. 3. The variables, such as height, amniotic fluid contamination, Bishop score, age, bone length, weight, and abdominal circumference, contributed markedly greatly to the model.

**Figure 4:**
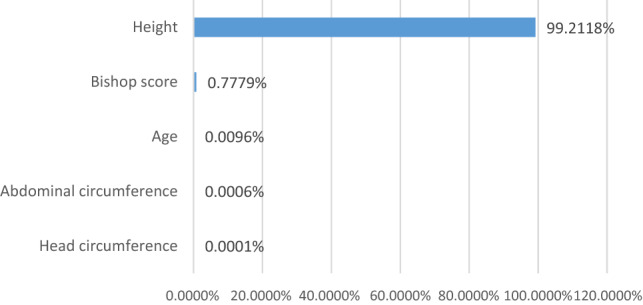
Ranking of the variables of logistic regression model of multiparas.

The distribution of the variables incorporated in the logistic regression prediction model of multiparas is shown in Fig. 4. The variables, such as height, Bishop score, age, abdominal circumference, and head circumference, contributed greatly to the model.

## Discussion

### Innovation of the research

In this study, the subjects were subdivided into the primipara and multipara groups, considering that different times of delivery had a significant impact on the outcome of induced labor, which was scientific and practical. The advantages of this approach were as follows: It improved the diagnosis and treatment level of young doctors and grass-roots hospitals and helped them individually in understanding the risk of induction labor treatment of pregnant women according to the results of predictive model analysis. This would guide the clinicians in choosing the mode of induced labor, improve the success rate of induced labor, and reduce the rate of cesarean section and perinatal mortality. It improved the credibility of doctors’ explanations to patients. Interestingly, when doctors recommend OT-induced labor, pregnant women and their families want to know the risk of treatment and the probability of successfully induced labor; however, the doctors can only provide uncertain subjective answers. With this model, we could accurately calculate the quantitative prediction results using the machine learning algorithm to provide a confident answer to the patient.

### The prediction performance of the model

In the primipara and multipara groups, the logical regression algorithm performed adequately, and the prediction result was obviously better than the other three algorithms. As a classical machine learning algorithm, it has a history of several decades. It has a fast training speed, is simple and easy to understand, and is suitable for binary classification problems. In the medical field, it is widely used in early diagnosis and risk prediction, such as “classification of Alzheimer’s disease based on regularized logic regression” and “risk prediction of postoperative hypoproteinemia.” In this study, the correlation between variables is complex, which requires high response speed and accuracy of the model. Therefore, it is more advantageous to establish the outcome model of OT-induced labor based on the logistic regression model. In this study, the dependent variables were divided into two categories: “induced labor success” and “induced labor failure” according to the actual delivery outcome. In the clinical verification of the model, the prediction accuracy of “induced labor success” was the highest in the two groups, up to 94.24% and 96.55%, respectively. It supports the decision of the risk of OT-induced labor treatment, and the high-risk population predicted by the model can further enhance the risk awareness of medical staff, thus preparing for the time of delivery by increasing staffing and anesthesia accordingly. The positive predictive value of “induced labor failure” was low in the model, which could be affected by the small number of training samples and maternal psychological factors, such as tension, fear, anxiety, and other emotions, that have a significant influence on OT-induced labor failure. However, since this is a retrospective study, the relevant data, such as maternal psychological state, cannot be obtained, thereby affecting the prediction accuracy of the model for “induced labor failure”^15^.

### Discussion on the related factors influencing the outcome of OT-induced labor

The results showed that the factors affecting the outcome of labor induced by OT were age, height, weight, amniotic fluid contamination, fetal head circumference, and thigh length Bishop score. This was consistent with the requirements of clinical medical staff for antenatal evaluation index and observation guide of OT, indicating that the OT-induced labor outcome model established in this study simulates clinical thinking, and the modeling results have high credibility.

#### Height, age, weight, amniotic fluid contamination, and Bishop score are critical variables in predicting the outcome of OT-induced labor, which is consistent with previous studies.

Several studies have shown a significant correlation between maternal height and the outcome of induced labor. Compared to short height, taller women have a higher success rate of induced labor. This might be related to the narrower entrance to the pelvis in women with short stature, which makes it difficult for the fetus pass through the pelvic entrance plane smoothly, resulting in dystocia and increasing the risk of cesarean Section^16^.

In this study, the proportion of height > 155 cm in the successful induced labor group was higher than that in the failed induced labor group, suggesting that height can be used as one of the indexes to predict the effect of induced labor; this finding was consistent with the results of previous studies^17^.

Because of the lack of oxygen in the womb, the brain cannot control the lower center that relaxes the anal sphincter. Under the pressure of uterine contraction, meconium is excreted, leading to amniotic fluid contamination.

In addition, the study showed that pregnant women with a history of vaginal delivery have a significantly increased probability of successfully induced labor during subsequent deliveries, which was consistent with the results of this study.

Together, in addition to Bishop score, the above factors can also be included in the evaluation system to further improve the prediction accuracy in the evaluation of clinical OT-induced labor.

#### Fetal bone length and fetal head circumference are new findings in this study related to the outcome of OT-induced labor, but there are no similar reports in the literature

Fetal bone length and fetal head circumference were the factors newly found in this study that were related to the outcome of OT-induced labor, which might be associated with the lateral reflection of fetal weight. Although the fetus’s weight was not included in this study, fetal bone length and fetal head circumference reflect this factor in the results of B-ultrasound. Presently, there is no consensus on the impact of fetal bone length and fetal head circumference on the outcome of OT-induced labor, and additional studies are required to determine the intercorrelation.

### Limitations of the study

1. Due to the influence of subjective factors, such as maternal psychological state, the accuracy of the prediction model in this study was affected.
2. This was a single-center retrospective study, which should be further verified by prospective studies using multiple places and large samples in the future.

## Conclusion

In this study, the outcome prediction models of OT-induced labor in primipara and multipara groups were constructed, and the variables, such as age, height, and amniotic fluid contamination, were included based on Bishop score, which greatly improved the prediction performance of the model. In the clinical application of the model, clinical medical staff could choose the corresponding model according to the actual situation of pregnant women to predict the effect of OT-induced labor. In future studies, the model can be embedded in the information system, which is expected to further develop into an active early warning system for medical staff while making clinical decisions.

## Data Availability

The datasets used in the current study are available from the corresponding author upon reasonable request.
